# Photosynthesis in variable environments

**DOI:** 10.1093/jxb/erv175

**Published:** 2015-04-27

**Authors:** Giles N Johnson, Tracy Lawson, Erik H Murchie, Christine Raines

**Affiliations:** University of Manchester, UK; University of Essex, UK; University of Nottingham, UK; University of Essex, UK

Photosynthesis is one of the most important biochemical processes, underlying almost all life. It is also one of the most vulnerable to environmental stress. Irradiance changes over the diurnal cycle is broadly predictable but, superimposed on that cycle, changes in cloud cover can cause order-of-magnitude variations in irradiance from second to second. The extent of cloud cover will change with the season and, depending on the shorter term weather patterns, giving periods of days to weeks of higher or lower irradiance. For example, the low crop yields in the UK in 2012 have been attributed to low levels of sunshine during the summer of that year. Rapid changes in irradiance can also arise due to sunflecks caused by the movement of leaves and the position of the sun relative to gaps in the canopy. Variation in shading will also affect the spectral quality of absorbed light.

Changes in light absorption will have direct effects on the ability of plants to generate ATP and NADPH. At the same time, changes in temperature will affect the ability of plants to use those products of electron transport. The lowering of temperatures will result in a lowering of the rate of enzyme-catalysed reactions. At higher temperatures, changes in the solubility of CO_2_ and O_2_ and in the specificity of Rubisco will alter the extent of photorespiration. Temperatures fluctuate diurnally, but also depend on weather patterns over days to months. Fluctuations in water supply, even over short time periods, can lead to variation in stomatal conductance causing the CO_2_ supply to fluctuate.

All of the above factors can lead to imbalances between light capture and light use by the photosynthetic apparatus. When light absorption exceeds the capacity for the energy to be fixed, damage is likely to occur, either directly to the photosynthetic apparatus (especially Photosystem II) or more widespread damage caused by the generation of reactive oxygen species, such as singlet oxygen, superoxide, and hydrogen peroxide. In view of this, it might be thought that plants are in constant danger of bleaching themselves to death. The fact that this does not occur, that most plants are able to tolerate and even thrive in a wide range of continually fluctuating environments, is explained by an array of regulatory mechanisms that optimize the photosynthetic apparatus to the conditions experienced. These mechanisms operate on time-scales ranging from minutes to weeks, with rapid responses involving the regulation of metabolic processes being followed by reengineering of metabolism through changing gene expression and protein concentrations.

Most work in plant biology focuses on plants at a steady state. At the 2014 Annual Meeting of the Society of Experimental Biology, there was a session specifically focusing on how plants respond to *fluctuations* in the environment, in particular, in relation to the effects of dynamic conditions on photosynthesis. Papers included in this focus section present an excellent overview of the state of research in this emerging field, with a range of different approaches being covered. **Schöttler *et al*.** focus on how different environments alter the composition of the thylakoid membrane, describing work in particular that uses spectroscopic approaches to understand the importance of different responses. The review by **Dietz** looks at work using a systems biology approach in order to understand the early events in the response of plants to changes in light, examining sensing and signalling pathways. **Kaiser *et al*.** examine functional responses to fluctuations while **Allahverdiyeva *et al*.** look at processes involved in preventing damage to the photosynthetic apparatus during stress. In addition, the review by **Retake *et al***. adopt a modelling approach to understand what the optimal solution is for plants to acclimate photosynthesis to different fluctuating conditions.

Together, these papers provide a good cross-section of the range of different experimental approaches being adopted to understand how plants can improve their photosynthetic performance in natural environments. If we are to increase crop yields to meet growing demands, improving performance under natural conditions will be an essential step, which current strategies are poor at targeting. Defining the important types of fluctuation that affect plant productivity and identifying strategies that allow plants to cope with those will be an important step in meeting productivity goals.

